# Reliability and Sensitivity of a Virtual Assessment Developed for Workplace Concussions: Protocol for a Method-Comparison Study

**DOI:** 10.2196/57663

**Published:** 2024-07-26

**Authors:** Keely Barnes, Heidi Sveistrup, Mark Bayley, Michel Rathbone, Monica Taljaard, Mary Egan, Martin Bilodeau, Motahareh Karimijashni, Shawn Marshall

**Affiliations:** 1 School of Rehabilitation Sciences Faculty of Health Sciences University of Ottawa Ottawa, ON Canada; 2 Bruyère Research Institute Ottawa, ON Canada; 3 Ottawa Hospital Research Institute Ottawa, ON Canada; 4 School of Human Kinetics Faculty of Health Sciences University of Ottawa Ottawa, ON Canada; 5 Systems and Computer Engineering Technology Carleton University Ottawa, ON Canada; 6 Kite Research Institute Toronto Rehabilitation Institute University Health Network Toronto, ON Canada; 7 Division of Physical Medicine and Rehabilitation Temerty Faculty of Medicine University of Toronto Toronto, ON Canada; 8 Department of Medicine, Division of Neurology Faculty of Health Sciences McMaster University Hamilton, ON Canada; 9 School of Epidemiology and Public Health University of Ottawa Ottawa, ON Canada; 10 Department of Medicine University of Ottawa Ottawa, ON Canada

**Keywords:** telehealth, virtual care, concussion, mTBI, mild traumatic brain injury, assessment, examination

## Abstract

**Background:**

Workplace mild traumatic brain injuries are frequently associated with persistent symptoms, leading to a reduction in productivity at work or even disability. People who sustain workplace injuries frequently need rehabilitation and support, and the challenges of delivering these services was heightened during the COVID-19 pandemic as injured workers had to be cared for remotely. Currently, clinicians are conducting both in-person and virtual (remote) concussion assessments; however, the measures that are being used to complete these assessments have undocumented psychometric properties.

**Objective:**

This study will document the psychometric properties of the clinical measures that are being used remotely and their ability to produce similar results to in-person assessments. Specifically, through this method-comparison study, we aim to (1) evaluate the sensitivity of the measures included in a virtual assessment toolkit when compared to an in-person assessment and (2) determine the interrater and intrarater reliabilities of the measures included in a virtual assessment toolkit.

**Methods:**

Patient participants (people living with acquired brain injuries) will attend two assessments (in person and virtual) at the Ottawa Hospital. The two assessments will be identical, consisting of the measures included in our previously developed virtual concussion assessment toolkit, which includes finger-to-nose testing, the Vestibular/Ocular Motor Screening tool, balance testing, cervical spine range of motion, saccades testing, and evaluation of effort. All virtual assessments will occur using the Microsoft Teams platform and will be audio/video-recorded. The clinician assessor and patient participant will complete a feedback form following completion of the assessments. A different clinician will also document the findings on observed videos of the virtual assessment shortly after completion of both in-person and virtual assessments and approximately 1 month later. Interrater reliability will be assessed by comparing the second clinician’s observation with the first clinician’s initial virtual assessment. Intrarater reliability will be evaluated by comparing the second clinician’s observation with their own assessment approximately 1 month later. Sensitivity will be documented by comparing the findings (identification of abnormality) of the in-person assessment completed by the initial clinician assessor with those of the second clinician assessor on the observation of the recording of the virtual assessment.

**Results:**

As of May 2024, we have recruited 7 clinician assessors and completed study assessments with 39 patient participants. The study recruitment is expected to be completed by September 2024.

**Conclusions:**

Currently, it is unknown if completing concussion assessments virtually produces similar results to the in-person assessment. This work will serve as a first step to determining the similarity of the virtual assessment to the matching in-person assessment and will provide information on the reliability of the virtual assessment.

**International Registered Report Identifier (IRRID):**

DERR1-10.2196/57663

## Introduction

### Background

A direct or indirect head impact leading to a concussion is a form of mild traumatic brain injury [1], which results in an alteration of brain functioning [2,3]. Most individuals who sustain a concussion experience rapid recovery within the first 2 weeks to 1 month following the injury. There are, however, instances where individuals continue to experience persistent symptoms after a concussion, with a reported occurrence of approximately 15%-20% [4]. These symptoms may persist beyond 1 year following the injury [3,5]. Factors such as premorbid characteristics (age, sex, concussion history), psychological factors, and injury-related factors (ie, severity of injury) are likely contributors to the persistence of the symptoms [6]. Postconcussion symptom presentation can vary widely, including cognitive deficits such as memory issues, vestibular deficits such as balance issues or dizziness, and ocular deficits such as blurred vision, among others [1,7-9]. There is a need for a multifactorial assessment approach due to the diversity in symptom presentation. Specifically, it is recommended to incorporate measures targeting each of the commonly experienced symptoms into a battery of tests [10].

There is evidence suggesting a growing documented incidence of concussion in the workplace context [11]. Kristman et al [12] reported that there are approximately 39-58 workplace concussions per 10,000 claims annually in Ontario, Canada [12]. Workplace concussions often lead to greater delays in return to work compared to injuries sustained outside of the workplace context [5,13]. It is estimated that roughly 60% to 90% of adults return to work within 6 months postconcussion injury, with the remainder continuing to be off work at this time point [5]. Compensation associated with a workplace injury may be a contributor to this delay in return to work [5,13].

Several factors can contribute to delays in return to work following concussion, including a lower level of education, more severe immediate symptoms post injury, and other injuries sustained [5]. In general, workplace concussions pose significant challenges. These include financial burdens that are felt by the health care system, employers, the Workplace Safety and Insurance Board (WSIB), and the workers themselves [14]. For the patient, lives are disrupted due to prolonged recovery times [13], resulting in the need for additional support from the WSIB for individuals presenting for specialized assessments.

The onset of the COVID-19 pandemic affected how specialized postconcussion assessments could be completed along with a need to switch to virtual care [15]. However, it is not known whether the measures being used by clinicians in virtual assessments are the same as those used for in-person assessments [16]. Additionally, the reliability of the virtual concussion assessments being conducted is unknown. Measures used in person that have shown strong psychometric properties may not produce the same or similar results when administered virtually [17]. Furthermore, the level of agreement between measures used in the in-person concussion assessment and those used in the virtual assessment has not yet been established. Currently, clinicians are continuing to complete both in-person and virtual concussion assessments; however, there is limited information on the measures used in the virtual assessment. It is important to gain an understanding of the properties of the clinical measures and their ability to produce similar results to the in-person assessment to inform the effectiveness of use in a virtual context [18].

The preparatory work for this study was initiated by developing a virtual assessment toolkit. Clinician surveys were used to identify relevant measures for assessing the physical domains of concussion [19]. A working group refined the measures feasible for virtual administration based on expert opinion. Focus groups then identified barriers and facilitators for virtual concussion assessments. The resulting virtual assessment toolkit was then used in a feasibility study to evaluate recruitment rates, procedure acceptability, and time to assess; the manuscript reporting these findings is currently being prepared for submission.

### Objectives

The objectives of this method-comparison study are two-fold: (1) to evaluate the sensitivity of the virtual assessment against the gold standard of the in-person assessment and (2) to determine interrater and intrarater reliabilities of the measures in the virtual assessment. This study will further contribute to the development of a virtual toolkit of assessments across four physical domains along with the evaluation of effort, specifically designed for the WSIB context.

## Methods

### Design

This study will follow a prospective method-comparison study design [17].

### Participants

#### Patient Participants

Eligible participants will be adults 18 years or older who have sustained an acquired brain injury (concussion or other form) and are being assessed by a physician, physician assistant, and/or physiotherapist at the Ottawa Hospital. In addition to people with concussions, people living with acquired brain injuries, including moderate to severe traumatic brain injury or hypoxic brain injury, will be recruited. In this study, we are assessing the consistency of physical examination findings; people with other forms of acquired brain injury such as severe traumatic brain injury more frequently have signs of central nervous system deficits, whereas these assessments are often normal during a postconcussion examination [20,21]. This inclusion is thus intended to ensure recruitment of participants with a broad range of identifiable deficits on all components of the assessment. Those unable to complete the study assessments and unable to speak English or French will be excluded from the study. Participant information will be documented, including demographics, functional status, environmental aids used, socioeconomic and work status, injury information, and information related to technology experience.

#### Clinician Participants

Clinicians with expertise in conducting assessments for people with acquired brain injuries will be invited to participate in this project. Demographic information for participating clinicians will be documented, including age, years practicing with patients who have an acquired brain injury, professional background (physiotherapist, physician assistant, physiatrist, other), and self-reports of competency with in-person and virtual neurological assessments.

### Sample Size

The target sample size of this method-comparison study is 60 patient participants. For the objective of assessing the sensitivity of the virtual assessment against the gold standard of in-person assessment, with a total of 60 participants, 50 (83%) of whom are expected to have an abnormal finding, and an estimated sensitivity ranging from 77% to 96%, the two-sided 95% CI around the estimated sensitivity will have a total width ranging from 8.7% to 25.3% (or margin of error 4.4%-12.7%). For the objective of assessing interrater agreement between two clinicians conducting a virtual assessment using the κ statistic, a sample size of 60 participants achieves 80% power to detect a true κ value of 0.89 using a one-sided test at the 5% significance level, assuming that 90% of participants are classified as abnormal; κ under the null hypothesis was specified as 0.5.

### Recruitment and Consent

#### Patient Participants

Purposive sampling methods will be used to recruit individuals living with acquired brain injuries with impairments spanning all domains of interest. The participants will be recruited through the Ontario Workers Network and relevant rehabilitation clinics at the Ottawa Hospital. Electronic medical records will be screened to identify eligible patients, who will then be called or approached in person for consent.

#### Clinician Participants

Clinicians will be recruited from the Ontario Workers Network and relevant rehabilitation clinics at the Ottawa Hospital to participate as clinician assessors in the study. Consent will be obtained over the telephone or face to face.

### Outcomes

The virtual toolkit of assessments will include the following outcome measures: the classification of abnormality (binary variable) on the finger-to-nose test, Vestibular/Ocular Motor Screening tool, balance testing, cervical spine range of motion, saccades testing, and evaluation of effort. The procedures outlined below provide details for these outcome measures, including the methods used for evaluating the sensitivity of the virtual assessment when compared to the in-person assessment and the interrater and intrarater reliabilities of the virtual assessment.

### Procedures

#### Training

Training of the assessing clinicians on the outcome measures will occur prior to commencement of patient-participant recruitment. A previously developed training manual [22] will be used to complete the training. Training will be uniform with the goal of standardizing clinician completion of the assessments.

#### Assessments

Data collection will occur in Research Electronic Data Capture (REDCap), a secure electronic data capture application. An assessing clinician will conduct two assessments: one in person and one virtual. Both assessments will be identical consisting of all measures from the virtual assessment toolkit (see Table 1). Both assessments will be completed at the Ottawa Hospital. When possible, assessments will occur on the same day with a brief rest period in between [23,24]. The order of assessment completion (in person vs virtual) will be randomized and counter-balanced. A random-numbers table will be generated and imported into REDCap and the randomization will occur through REDCap. In addition to the assessments identified in Table 1, clinicians will document adverse events (if any) and will record findings from the assessments on a paper copy, which will be directly input into REDCap by a research team member.

For the virtual assessment, the patient participants will be located in a separate room from the assessing clinicians within the Ottawa Hospital. All patient participants will use the same computer and will be provided with technical support from a research team member as needed. The virtual assessments will be conducted in the clinical environment to standardize as many components of the assessment as possible, including computer screen, lighting, image size, and room setup. During the virtual assessment, several safety measures will be implemented: (1) a research team member will be present in the room throughout and (2) the patient participant will stand in front of a wall/bed/chair or with their back at a corner during the vestibular assessments in case of loss of balance. All the virtual assessments will occur through the Microsoft Teams platform. The study assessments will be audio/video-recorded using the record function on Microsoft Teams.

Where possible, both assessments will be completed on the same day. It is estimated that a portion (~5%) of participants may be unable to complete both the in-person and virtual assessments on the same day, either due to lack of time or inability to tolerate a second assessment as a result of symptom aggravation (eg, headaches, dizziness, nausea, sensitivity to noise and light). For these participants, the second assessment (virtual or in person) will be scheduled for completion within 1 week of the initial assessment.

Following completion of the assessments, the patient participants and clinician assessors will complete a feedback form. Participants will complete a feedback form (see Multimedia Appendix 1) related to completion of the virtual assessment, the environmental setup, and confidence in the assessment findings.

**Table 1:** Outline of measures included in the virtual assessment toolkit to be completed in both in-person and virtual assessments^a^.

Domain	Measures	Documentation of findings by clinicians
Neurological examination	Coordination: finger-to-nose test	Abnormal (hesitation, tremor, over- or undershooting [25])/normal
Vestibular	VOMS^b^	Change in symptoms (≥2 point increase indicates abnormality [26]); average distance for 3 trials of near-point convergence in centimeters (≥5 cm convergence indicates abnormality [26])
Vestibular	Balance (feet together, single-leg stance, tandem stance) with eyes open and eyes closed for 20 seconds each	Abnormal (unable to complete 20-second balance [22])/normal
Oculomotor	Saccades	Abnormal (observe saccade initiation, range of motion and conjugacy, speed, accuracy, intrusions, or oscillations [27])/normal
Cervical	Range of motion (flexion, extension, right and left lateral flexion, right and left rotation)	Estimated angles for each recorded abnormality (determined by comparison to norms in people aged 20-59 years: flexion=50°-72°, extension=58°-77°, lateral flexion=37°-47°, rotation=67°-81° [28])
Effort	Rating scale	Full use of effort documented as yes or no (clinician discretion); rating scale documented from 0 (no effort) to 10 (maximum effort) (clinician discretion)

^a^For a complete presentation of the instructions used to administer the measures, see Johnston et al [22].

^b^VOMS: Vestibular/Ocular Motor Screening.

#### Observation and Rating of Audio-Video Recordings

**Figure 1:**
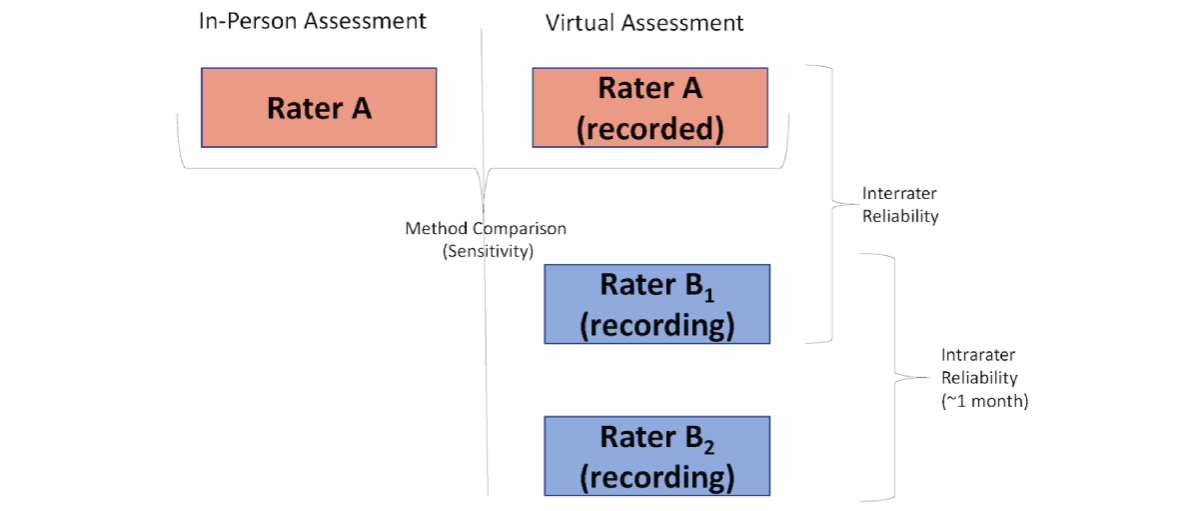
Assessment procedures (adapted from Russell et al [17]).

A second clinician (rater B in Figure 1 [17]) will complete two ratings of the recording of the virtual assessment (in a randomized order for participants) at 1-month intervals. Clinicians will record findings from the assessments on paper or electronic copies, which will be input directly into REDCap by a research team member. See Figure 1 [17] for an outline of study assessment procedures.

### Analysis

#### Assessments Overview

Patient and clinician participant characteristics will be descriptively analyzed. IBM SPSS (version 28) will be used to calculate the sensitivity of virtual assessments versus in-person assessments, along with the κ values (interrater and intrarater reliabilities) of the virtual assessments (see Figure 1 for comparisons).

#### Method-Comparison (Sensitivity) Analysis

The sensitivity of the virtual assessment compared to the gold-standard in-person assessment (rater A in person vs rater A virtual) will be determined, where the assessment of the uncertain classification of the measures used virtually will occur against the gold standard or “true” classification, which is the in-person assessment in this study. The findings of the initial clinician assessor (rater A) that completed the in-person and virtual assessments will be compared to determine the sensitivity of the measures administered virtually. In this case, sensitivity (true positives divided by the sum of true positives and false negatives multiplied by 100) refers to the ability of the virtual measure to identify a deficit or positive finding when the in-person measure identifies the deficit or positive finding. Values closer to 100% indicate greater sensitivity.

#### Reliability

To calculate the interrater reliability of the virtual assessment, the findings of rater A (virtual assessment) will be compared to the findings of rater B_1_ (initial observation of the virtual assessment recording). To determine the intrarater reliability of the virtual assessment, the findings of rater B_1_ (initial observation of the virtual assessment) will be compared to the findings of rater B_2_ (second observation of the virtual assessment) approximately 1 month after the initial observation. Estimates of reliability will be calculated using the κ statistic (classification of abnormal vs normal) for all measures included in the toolkit.

#### Adverse Events

Adverse events, including the type and severity, will be summarized by assessment type (in person or virtual).

### Ethical Considerations

Ethics approval was obtained from the Ottawa Health Sciences Network Research Ethics Board (20230311-01H), the Bruyère Research Ethics Board (M16-22-006), and the University of Ottawa Board of Ethics (H-06-23-9348). All potential risks and adverse consequences will be relayed to participants verbally and all participants will provide verbal consent over the telephone or informed consent in person prior to their scheduled appointment. A wet-ink signature will be obtained from all participants on the day of their scheduled assessment. Privacy and confidentiality will be maintained by using deidentified data. Patient participants will be provided with a CAD $30 (~US $22) gift card and parking voucher as a token of appreciation for their participation in the study. 

## Results

As of May 2024, we enrolled and completed both the in-person and virtual assessments for 39 patient participants. We have recruited 7 clinician assessors. Recruitment and testing are expected to be completed by September 2024.

## Discussion

### Projected Significance

This study will establish the reliability and sensitivity properties associated with 5 physical components of the virtual concussion assessment. There is an identified gap in current knowledge with limited research conducted on the reliability and comparability of virtual concussion assessments in comparison to in-person assessments. While virtual care has demonstrated to be a valuable approach to concussion assessment and management [29,30], there is a need for additional information regarding the psychometric properties of outcome measures. This information is essential for instilling confidence in the administration of these measures in a practical setting. Virtual approaches to care have been used for many years; however, the COVID-19 pandemic significantly increased their use in an effort to reduce hospital visits [15]. While the COVID-19 pandemic initiated this increased use of virtual care, some assessments are continuing to be offered virtually. Using measures with acceptable properties in a virtual context is important in practice to ensure accurate identification of problem areas on virtual assessments so that treatment interventions can be appropriately directed [31]. Despite measures showing adequate properties when administered in person, they may produce different results when administered in a virtual context [17,32], thereby providing a rationale to carry out this work. The aim of completing this method-comparison study is to obtain preliminary information on the virtual assessment in relation to the WSIB context.

### Strengths and Limitations of the Methodological Approach

The strengths of this method-comparison approach include the use of commonly adopted technology and software for virtual brain injury assessments in a practical setting. Further, including a wide range of acquired brain injury severities in our sample ensures the inclusion of abnormalities spanning all relevant domains. This will make the findings not only applicable to current practice but also potentially generalizable to different types of acquired brain injury.

We plan to assess the virtual evaluation in a controlled hospital setting. Moving forward, it would be beneficial to evaluate the virtual assessment in environments that are less standardized such as patients’ homes or community clinics where physician specialists are in a different location than the patient.

Finally, due to constraints in clinic scheduling, a methodological decision was made to have the same clinician (rater A) conduct both the in-person and virtual assessments with the participants. In attempts to address this concern, the order of study assessments (in person and virtual) will be randomized and counter-balanced.

### Conclusion

This method-comparison study is an important endeavor considering the limited exploration of virtual concussion assessments in the literature despite their increasing use in practice. This study aims to determine whether virtual and in-person assessments yield similar results in terms of the identification of abnormality in common physical domains of a concussion assessment. The study will also assess the interrater and intrarater reliabilities of the virtual assessment, contributing valuable insights to the field of concussion rehabilitation.

## Appendix

Multimedia Appendix 1 Feedback forms.

Multimedia Appendix 2 Peer-review reports from the Strategic Research - Workplace Safety and Insurance Board (WSIB) (Ontario, Canada).

## Abbreviations

REDCap: Research Electronic Data Capture
WSIB: Workplace Safety and Insurance Board
