# Supplementary material for: Reliability and Sensitivity of a Virtual Assessment Developed for Workplace Concussions: Protocol for a Method-Comparison Study
Source: JMIR Res Protoc. 2024 Jul 26;13:e57663. doi: 10.2196/57663 (PMC11316157; doi:10.2196/57663)
Supplement: Multimedia Appendix 1 [file resprot_v13i1e57663_app1.pdf]

Patient-Participant Feedback

1. Do you have any feedback about the environmental set-up of the virtual assessment (screen size, lighting, space, etc.)?

2. Do you think you your clinician obtained the same information in the virtual assessment when compared to the in-person assessment?

3. I feel confident in my clinicians' findings on the virtual assessment:

- ○ Strongly Disagree
- ○ Disagree
- ○ Neutral
- ○ Agree
- ○ Strongly Agree

4. I feel confident in my clinicians' findings on the in-person assessment:

- ○ Strongly Disagree
- ○ Disagree
- ○ Neutral
- ○ Agree
- ○ Strongly Agree

5. Do you have any other feedback regarding the virtual assessment?

Clinician Feedback

1. Do you have any feedback about the environmental set-up of the virtual assessment (screen size, lighting, space, etc.)?

2. Do you think you obtained the same information in the virtual assessment when compared to the in-person assessment?

3. I feel confident in my findings on the virtual assessment:

- ○ Strongly Disagree
- ○ Disagree
- ○ Neutral
- ○ Agree
- ○ Strongly Agree

4. I feel confident in my findings on the in-person assessment:

- ○ Strongly Disagree
- ○ Disagree
- ○ Neutral
- ○ Agree
- ○ Strongly Agree

5. Do you have any other feedback regarding the virtual assessment?
